# Grounded Theory-Based User Needs Mining and Its Impact on APP Downloads: Exampled With WeChat APP

**DOI:** 10.3389/fpsyg.2022.875310

**Published:** 2022-06-14

**Authors:** Tinggui Chen, Chu Zhang, Jianjun Yang, Guodong Cong

**Affiliations:** ^1^School of Statistics and Mathematics, Zhejiang Gongshang University, Hangzhou, China; ^2^Academy of Zhejiang Culture Industry Innovation & Development, Zhejiang Gongshang University, Hangzhou, China; ^3^Department of Computer Science and Information Systems, University of North Georgia, Oakwood, GA, United States; ^4^School of Tourism and Urban-Rural Planning, Zhejiang Gongshang University, Hangzhou, China

**Keywords:** online reviews, Grounded Theory, user demand mining, quantile regression, APP downloads

## Abstract

Software development is an iterative process from designing to implementation, and to testing, in which product development staff should be closely integrated with users. Satisfying user needs effectively is often the pain point for developers. In order to alleviate this, this paper manages to establish the quantitative connection between users' online reviews and APP (Application Program) downloads. By analyzing user online comments, companies can dig out user needs and preferences. This could benefit them by making accurate market positioning of their APP products, and therefore iteratively innovating products based on user needs, which hopefully will increase the volume of APP downloads. This paper regards WeChat APP during 47 updates periods as the research object. Based on Grounded Theory, user needs are extracted after data cleaning. Next, by using semantic analysis and word frequency analysis, we are able to obtain the implicit feedbacks such as emotion tendency, satisfaction and requirements lie under online reviews. Then, we construct a quantile regression model to study the impact of users' online reviews on downloads based on the influencing factors we extracted so as to provide a decision basis for enterprises to iteratively update their products. Results show that: (1) Generally speaking, needs of WeChat users mainly focus on performance, reliability, usability, functional deficiency, functional insufficiency, and system adaptability; (2) For those APP versions with relatively fewer downloads, user needs are mostly about functional deficiency, followed by functional insufficiency, performance, usability, and system adaptability. At this stage, it is found out that users' emotion tendency and user satisfaction significantly affect the volume of downloads; (3) When the volume of APP downloads is moderate, the user needs are functional deficiency, functional insufficiency, and system adaptability. While under this circumstances, users' star ratings have a significant impact on downloads; (4) In addition, when the volume of App downloads is high, user needs are performance, usability, and system adaptability. Our methods effectively extract users' requirements from online reviews and then successfully build up the quantitative connection between the implicit feedbacks from those requirements and APP downloads.

## Introduction

Many studies shown that online review data posted by users on the Internet has important direction guidance and strategic value for product development and iteration. This is because online comments are rich in user needs (Pagano and Maalej, 2013), and existing comments will likely to affect other users' consumption behaviors (Kim et al., 2015). Therefore, this paper proposes two research questions: one is how to mine user needs from online reviews; the other is how online reviews affect users' consumption behavior. Facing the fast-growing and fiercely competitive APP development industry, how to quickly and accurately obtain user needs and make the functionalities of their product match user needs is enterprises' urgent problem. Based on this, mining user needs from online reviews and providing decision-making basis have important research value for enterprises to upgrade and maintain APP products. This paper focuses on two problems, user requirements mining from the online reviews, and studying the influence of those requirements from online reviews on users' consumption behavior. Hopefully, user needs mining would bring suggestions to APP development and iterations and eventually improve user experience.

In the past, development enterprises collected customers' opinions through offline market surveys and interviews to obtain user needs, but they often failed to accurately capture heterogeneous market needs. Meanwhile, the amount of online comment data is often very large, so it is a difficult problem to select an appropriate method to mine user needs accurately and comprehensively. In addition, in the iterative update process of APP, user needs change correspondingly, thus it is a huge challenge for enterprises whether each update of APP products can meet user needs. In fact, the degree of satisfaction is directly related to the user's download behavior of APP products. Usually, if the users' desired functionality is not updated or the bug that needs urgent fix is not improved, users will generally uninstall the APP and choose alternatives that will meet their needs. As it turns out, the company will face the reduction of APP downloads and the loss of users. Thus, it indicates that APP downloads can be used to measure the satisfaction degree of user needs. Previous studies had shown that online reviews contain information about user needs, users' satisfaction and evaluation for product, and users will be affected by these factors in other users' online reviews when downloading APPs. In addition, empirical studies have shown that online reviews will affect users' consumption behavior. Based on this, in this paper, user needs will be mined from online reviews, and the relationship between users' online reviews and APP downloads will be studied to provide scientific decision-making basis for enterprises to iterate and update products. At present, there are relatively few studies based on online reviews that analyze the relationships between APP downloads and users' online reviews. Most studies focus on the analysis of the content length of online reviews, users' emotional tendencies, and scores or rankings in the APP store. For example, Burgers et al. (2016) found that the positive valence (emotion) of online reviews was positively correlated with APP downloads. Through Spearman's correlation analysis, Wang et al. (2016) found out that there was a strong correlation between APP name scores, APP rankings and APP downloads. In fact, online review data contains a lot of valued information, which not only reflects users' emotional inclination and satisfaction with APP products, but also contains valuable user needs information. Users will consider all kinds of information about products in online reviews before making a purchase (Logrieco et al., 2021). Based on the above analysis, this paper takes WeChat APP as an example. Firstly, we dig out user needs from online comments based on Grounded Theory. Secondly, we build a quantile regression model, and use the volume of downloads as a measurement of user consumption behavior to study the impact of users' online review on APP downloads. Finally, we visualize user needs based on the quantile regression results and provide decision-making suggestions for companies to better match user needs when iteratively updating products.

This paper uses Grounded Theory to mine a relatively comprehensive user needs from online reviews and uses quantile regression model to discuss the impact of users' online review on APP downloads. It mainly makes contributions from the following aspects: first of all, when we use Grounded Theory to mine user needs, we combine the needs theory of system engineering, which will make the needs theory system we build more widely in the coverage of needs, and there is no research on such combination at present. Secondly, when we study the relationship between online reviews and APP downloads, we not only take users' emotion tendency and user satisfaction under consideration, but also generate user needs mined from online reviews as an explanatory variable (attention to user needs) into the regression model, which enables us to comprehensively consider various factors when studying the impact of online reviews on APP downloads. It fills in the research gap since previous study mostly focus on the impact of comment length and user sentiment on downloads.

The structure of the paper is organized as follows: Section 2 is a literature review; Section 3 is the research framework of this paper; Data Acquisition and Preprocessing (or Preparing) explores user needs in online reviews based on Grounded Theory; The Impact of User Online Reviews on WeChat Downloads uses quantile regression model to explore impact of users' online reviews on APP downloads so as to provide a scientific basis for the decision-making of product iteration and update; Conclusions makes conclusions of the full paper and prospects for future work.

## Literature Review

The popularity and convenience of mobile devices make customers spend more and more time on mobile devices, and enterprises are launching more and more branded mobile APPs to reach and attract new and old customers. By the end of June 2021, 3.02 million APPs had been released on app stores in China's domestic market, according to monitoring data from the Ministry of Industry and Information Technology. However, after the quantities and types of APP software become saturated, the mismatch between functionalities and user needs is gradually severe, resulting in the loss of users of related products and the decline of product stickiness. Unfortunately, this phenomenon gradually becomes normal. For example, Baidu Post Bar suffered a serious user loss from 2014 to 2016. In December 2014, the total number of users covered by Baidu Post Bar was 179.41 million, and the total number of visits was 1,115.4 million. However, by December 2016, the total number of people covered by Baidu Post Bar was 111.35 million, and the total number of visits was 518.79 million (Qiao, 2019). Further investigation illustrated that it is because the product functions (numerous advertisements, chaotic management of post bar, uneven content quality, etc.) and user needs (strong topic, efficient communication, high content quality, less advertising, etc.) do not match (Qiao, 2019). On the contrary, the reason for the rapid development of Tik Tok, a short video platform, has much to do with its clear understanding of user needs. According to the survey, 85% of Tik Tok users are under the age of 24, and most of them are from the first and second-tier cities (Wu, 2017). Tik Tok caters to these people's curiosity and personalized needs from the aspects of short video community, creative shooting, beauty and music, thus topping the list in the short video APP industry (Wu, 2017). Moreover, polarity analysis studies showed how these videos have a strong playful character (Hu and Liu, 2004; Nouwen, 2021). In the traffic era, in order to prevent traffic loss (user loss), WeChat begin to add new functionalities such as “Channels” and “live broadcast” and other short video functionalities, which are the embodiment of enterprises beginning to honor user needs (Ceron et al., 2014). For developers, it is of great significance to listen to the feedbacks and needs of end users for software design and optimization (Qiao, 2019). Based on this, in this section, we analyzed the literature from the following two aspects in Table 1: (1) Comments contain a lot of content about user needs; (2) Online reviews can influence consumer behavior.

**Table 1:** Prior works and findings.

**Domain**	**Authors**	**Related works**	**Research findings**
Comments contain a lot of content about user needs	(Boyd et al., 2019)	Semantic analysis of online comments.	Comments generated by online users are very helpful for product development.
	(Martin et al., 2016)	Literature review method.	Development engineers extract bug reports and feature requests from reviews.
	(Palomba et al., 2015)	A study on how developers addressed user reviews to increase their APP's success in terms of ratings.	Developers implementing user needs in user reviews are rewarded in terms of APP ratings.
	(Pagano and Maalej, 2013)	They analyzed over one million reviews from the Apple APP Store.	Reviews typically contain multiple topics, such as user experience, bug reports, and feature requests.
	(Vasa et al., 2012)	They analyzed 8.7 million reviews from 17,330 APPs.	Ratings and reviews add value to both the developer and potential new users.
	(Lukyanenko et al., 2016)	They analyzed the challenges and opportunities associated with Participatory Design in User-Generated Content.	This feedback in online reviews can represent the “voice of the user” and be used to drive the development of the APP to improve the upcoming version.
	(Lee, 2009)	They used machine learning to automatically identify user needs from online comments.	They visualized the competitive landscape by mapping existing products in terms of the user needs that they address.
	(Palomba et al., 2017)	They analyzed the structure, semantics, sentiments of sentences contained in user reviews.	Extract useful (user) feedback from maintenance perspectives and recommend to developers changes to software artifacts.
Online reviews can influence consumer behavior.	(Hao, 2010)	They studied the impact of the emotional polarity of online reviews on consumers' purchase behavior.	Reveal the realistic relationship between online comments and consumers' overall purchase behavior and its general law over time.
	(Zhang and Xu, 2012)	They investigated the impact of microblog reviews on consumers' purchase behavior.	Microblog positive comments have a significant impact on consumers' perceived economic value and functional value.
	(Ma, 2017)	Through text analysis and empirical analysis to verify the impact of online comments on consumers' car purchase behavior.	Online comments on appearance, performance and comfort have a positive impact on consumers' purchase behavior, and the influence of appearance and performance is higher than that of comfort
	(Lu and Hu, 2019)	A regression model was established to analyze the effects of user reviews on the APP downloads.	Online comments have an impact on users' download behavior.
	(Xiong, 2014)	They studied the impact of online comment interpretation types on online consumers' purchase intention.	The type of positive interpretation has a positive impact on the perceived usefulness of online comments, while the type of negative interpretation has no significant impact.
	(Chatterjee, 2001)	They examined the effect of negative reviews on retailer evaluation and patronage intention.	Retail consumers will be less willing to buy when they see negative WOM (word-of-mouth).
	(Vermeulen and Seegers, 2009)	This research applied consideration set theory to model the impact of online hotel reviews on consumer choice.	Exposure to online reviews enhances hotel consideration in consumers.
	(Ju, 2017)	The data of online reviews and downloads of mobile applications were collected, calculated, analyzed.	The number and score of online comments have a significant positive impact on the download of mobile applications.

All in all, previous studies (Lee, 2009; Vasa et al., 2012; Pagano and Maalej, 2013; Palomba et al., 2015, 2017; Lukyanenko et al., 2016; Martin et al., 2016; Boyd et al., 2019) all showed that online reviews contain valuable information about user needs. This provides a powerful auxiliary support for the research of mining user needs from online reviews. In addition, the researchers also found that online reviews have a certain impact on consumer behavior. Based on the analysis of literature on the impact of online reviews on users' consumption behavior (Chatterjee, 2001; Vermeulen and Seegers, 2009; Hao, 2010; Zhang and Xu, 2012; Xiong, 2014; Ju, 2017; Ma, 2017; Lu and Hu, 2019), we find that needs information in online reviews also has an impact on users' consumption behavior. However, researchers in this field mostly studied the influence of online comments on users' consumption behavior from the perspective of the length of online comments, users' emotional tendency of comments in online reviews, and users' star rating in online reviews. Based on this, this paper makes up for the shortcomings of the above research, and studies the relationship between needs information, user emotional tendency, user star rating and downloads in online reviews. Finally, we find that quantile regression model, traditional multiple regression and correlation analysis are often used by researchers to study the impact of online reviews on APP downloads. Therefore, we will use quantile regression and multiple regression to study the impact of online reviews on APP downloads and compare the advantages and disadvantages of different methods.

## Research Framework

Whether the iterative update of the product can match user needs will affect his/her final download behavior. Therefore, before upgrading products, companies need to fully understand the user needs and incorporate them into the APP development plan. Digging out users' interests and concerns from APP online comments is an important way to obtain user needs. However, due to the large amount of data and uneven quality of current user online comments, how to dig out valuable user needs information from the massive user comment data is a key issue to be solved urgently.

**Figure 1:**
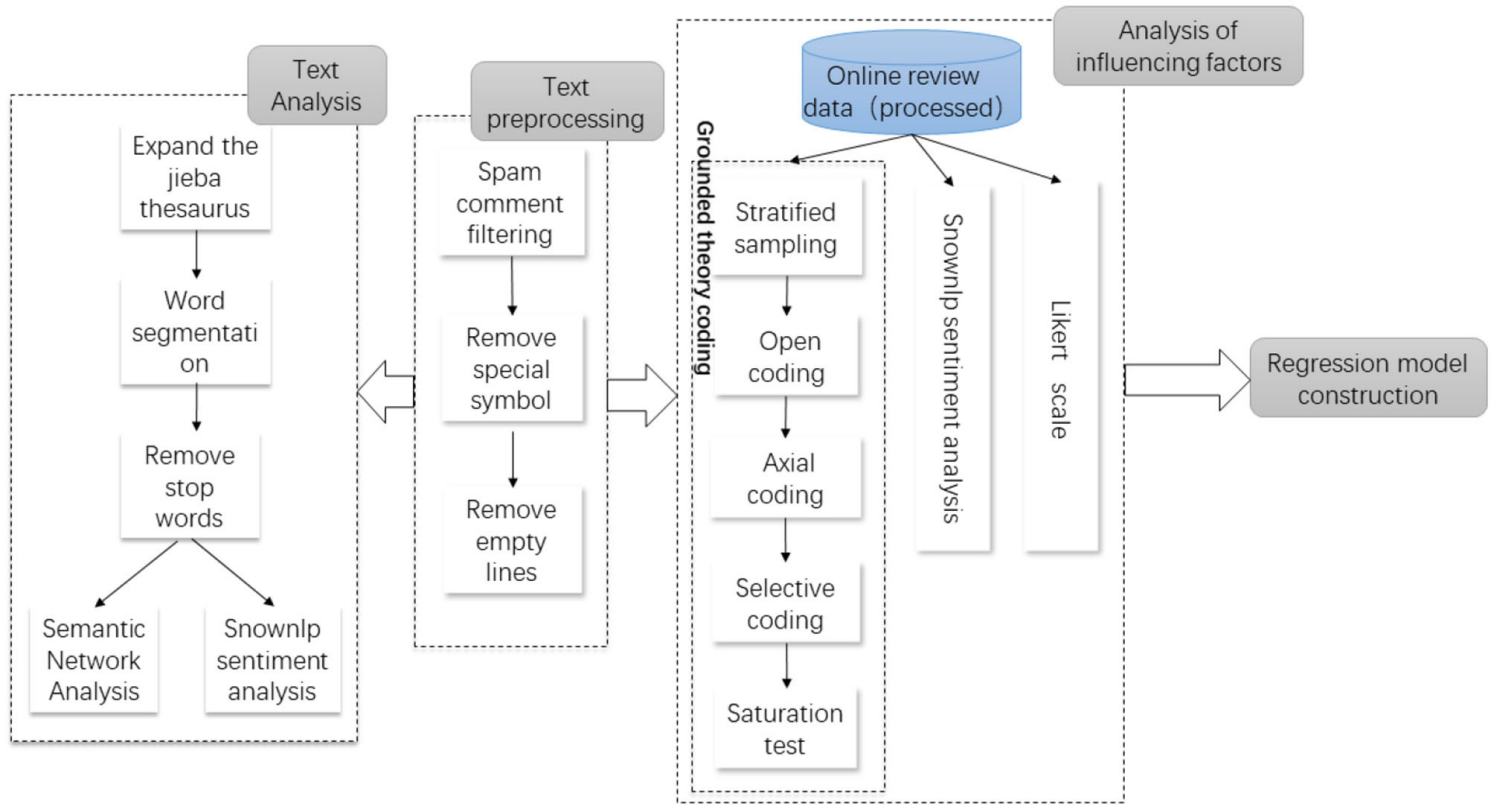
Research framework.

In this paper, we take WeChat APP as the research object, and obtain online comment data of its 47 updated versions through the KuChuan data platform and Weibo platform. First, we use Python for text preprocessing of comment data, including filtering spam comments, deleting invalid comments and empty lines. Then, stratified random sampling is carried out for the comments, and Grounded Theory is used to conduct open coding, spindle coding, selective coding and theoretical saturation test for the sampled text comments. User needs were quantified by text analysis method and users' emotional inclination and user satisfaction in online reviews of online comments were calculated. Finally, in order to study the influence of online reviews on consumer behavior, a quantile regression model was established for APP downloads and user needs (after quantification), users' emotion tendency and user satisfaction. The research idea of this paper is shown in Figure 1.

## Data Preparation

As the social chat APP with the largest number of users and the highest user stickiness in China, WeChat attracts a greater amount of attention and triggers heated discussion among netizens every time it releases an update version. According to the 2020 Financial Report of Tencent, by the end of 2020, monthly active users of WeChat in China have reached 1.225 billion, accounting for about 87.5% of China's total population (China's total population of 1.45 billion in 2020), which can be regarded as the most popular APP in China. Xiaolong Zhang, Senior Executive Vice President of Tencent and President of WeChat Business Unit, stated in the “2021 WeChat Open Class PRO”: every day, 1.09 billion users open WeChat, 330 million users make video call, 780 million users enter Moments, 120 million users release information on Moments, including 670 million photos, 100 million short videos, 360 million users read official accounts' articles, and 400 million users use mini programs. It can be seen that the users' stickiness and activity of WeChat APP are very high. However, every update of the WeChat APP version will cause a huge amount of discussion. According to data from the Weibo platform, some functional improvements, updates, such as changes to the WeChat ID, Tickle, and other functional topics discussed over 100 million times. At the same time, for APP development companies, in addition to user downloads, user needs preferences are also important. Online user reviews of products can well reflect the user's various functional and non-functional requirements for the product, as well as the emotion tendency of the product. For example, as for the comment “Chat history pictures these cannot expire in seven days,” through combining semantics and context, we can find that under this comment lies the user needs for extending the term of validity of chat pictures, which is a function to be improved. Also, as for the comment “Cannot open the pushed information,” we can learn that information hidden in this comment is that user needs stable features. Only after enterprises fully and accurately understand the user's product needs and preferences, can the developed products be more popular. Meanwhile, the downloads will increase accordingly. Based on the above discussion, reviews are of great important research value for the user needs mining of WeChat APP in the Chinese market.

### Data Acquisition and Preprocessing

Online comment data of WeChat APP researched in this paper are acquired from the Kuchuan data platform (https://www.kuchuan.com) and Weibo platform (https://mweibo.com). The Kuchuan data platform directly provides online comment download channel of various WeChat APP users. Since the update cycle and content of the iPhone Operation System (IOS) and Android Operation System (AOS) are different, user online comment data should be downloaded separately. After careful selection, Python is used to crawl user online comment data from the Weibo platform, including user comment time and mobile phone model, so as to facilitate later classification of comment data according to cycle and system. Since the online comment data in the APP before 6.2.5 version is too small, and the analysis conditions are not available, a total of 47 versions of the comment data after 6.2.5 (6.3.5–8.0.9) versions are selected. The duration is from December 29th, 2016, to July 29th, 2021, with a total of 1,109,972 pieces. Since there are many blank lines, repeated comments, special symbols, and irrelevant comments in the original data, this paper uses Python to remove special symbols and irrelevant comments and establish a loop statement to remove blank lines and repeated comments. After data cleaning, there are 696,801 valid user online comments.

### Mining User Needs

Before the research, this paper firstly analyzes and summarizes the previous methods of mining user needs based on online comments, as shown in the Table 2.

**Table 2:** Review of methods for mining user needs based on online comments.

**Domain**	**Authors**	**Method or model**	**Research work**
User needs mining based on online reviews	(Wang et al., 2020b)	-	Users make comments on products in the application market from different dimensions, which contain their needs for improving APP software.
	(Jang et al., 2017)	Latent Dirichlet Allocation Model	They used Latent Dirichlet Allocation Model to mine users' opinions from their online comments and provided a basis for management decision-making.
	(Xia et al., 2016)	K-means Clustering	A topic mining algorithm based on K-means clustering is applied to news comments.
	(Zhao et al., 2020)	Word Frequency Analysis and Manual Definition Methods	They selected candidate words according to the ranking of word frequency statistics. At the same time, he combined manual definition methods to determine the required keywords, and further mined user needs based on the keywords.
	(Liu et al., 2019)	Cluster Analysis and Multidimensional Scale Analysis	They used Chinese word segmentation and data analysis tools to realize word frequency statistics based on online comments and used statistical software to conduct cluster analysis and multidimensional scale analysis to classify product features and dig out potential user needs.
	(Adomavicius and Kwon, 2007) and (Lakiotaki et al., 2011)	Product Attribute Rating	They believed that users' scores for multiple attributes of a product contained more information about user needs than users' scores for a single product.
	(Kumar and Sebastian, 2012)	A Theory of Retrieving Vast Amounts of Information and Mining User Opinions	They proposed a theory to mine user needs from online reviews by retrieving relevant data from the vast amount of available comment information and then mining user opinions.
	(Han and Moghaddam, 2020)	Deep Language Model (BERT) and Machine Translation Algorithm	They proposed an efficient and extensible method for automatically and massively capturing attribute-level user requirements. This method was based on deep Language Model (BERT) to extract attribute, description and emotion words from online comment corpus. Also, machine translation algorithm was used to extract user needs expression of predefined part-of-speech combinations. Finally, the performance and feasibility of the method were proved by the empirical analysis of clothing and footwear.
	(Wang et al., 2020a)	Convolutional Neural Network	They proposed a solution based on convolutional neural network to map product reviews to product specifications. This method could well adapt to the mapping of customer requirements to product specifications in natural language.
	(Na and Zhong, 2013)	Natural Language Motion Analysis Technology and Constructing Fuzzy Inference Rules Based on Product Attributes	They developed a system to mine the display attributes and implicit attributes of products from online reviews, and established that the system could identify the emotions of consumer evaluations by using natural language emotion analysis technology and constructing fuzzy inference rules based on product attributes.
	(Semsar and Shirehjini, 2017)	Constructed a Web-based Intelligent 3D Simulator Experience Environment	Based on network experiments, they collected data from a large number of online participants and constructed a web-based intelligent 3D simulator experience environment to detect and respond to user needs, actions, behaviors and feelings.
	(Xu et al., 2019)	Long and Short-term Memory (LSTM)	They used long and short-term memory (LSTM) as hidden layer neuron and introduced attention mechanism to obtain information from text sequence and understood user comment text, so as to mine user needs.
	(Xu et al., 2017)	Text Mining to Connect Users' Online Comment Texts with User Experience	They used methods such as text mining to connect users' online comment texts with user experience, helping developers better understand customers' needs through user-created content.
	(Wang et al., 2018)	Sentiment Analysis and Regression Analysis	They conducted sentiment analysis and regression analysis on users' online comments to study how product attributes affected customer satisfaction, thus helping enterprises analyze user needs.
	(Austin et al., 2014)	Grounded Theory	Use Grounded Theory to iteratively encode the text of these reviews, identifying specific themes for urgent care, and thus providing a new strategy for assessing patient-centered quality in emergency care.
	(Ling and Gang, 2007; Yang and Lu, 2007)	Grounded Theory	in China tried to apply Grounded Theory method to tourism research and explore tourist behavior characteristics.
	(Lu et al., 2013)	Grounded Theory	analyzed the tourism reviews of three famous budget hotels (such as HOME INN, hanting Express, JINJIANGINN). Ctrip, a major online travel agency in China, tried to construct the dimension of tourists' online attention to budget hotels by applying Grounded Theory.

As for user needs mining based on online reviews, the above literature mainly uses LDA topic model, word frequency analysis, neural network, K-Means Clustering and other methods to mine user needs from online reviews. The LDA model does not have a good classification effect on short texts,the reason is that the LDA model mines the co-occurrence law between words. If the comment is very short, it is not conducive to the statistics of the co-occurrence law of words. Cluster Analysis is not effective in mining user needs from online reviews, this is because online comments are generally short texts with an average length of 10–15 words, which are highly colloquial and lack of grammatical rules. It is easy to make mistakes in word segmentation. Besides, TF-IDF algorithm (premise of using K-Means Clustering) simply reflects the importance of a word by its frequency, which is not comprehensive enough and thus K-Means Clustering is easy to fall into local optimization in the clustering process (Pan et al., 2011). Grounded Theory was developed by Barney and was a scientific approach proposed by Glaser and Strauss (1968) in 1967. It is defined as a qualitative research method that uses a set of systematic processes to develop an inductive and derived Grounded Theory approach to phenomena, the key goal of which being deeper analyzing the data (Charmaz, 2006). Grounded Theory research based on text review has been applied in many fields. Combined with semantic analysis in user needs mining, theoretical saturation test shows that user needs mining by Grounded Theory is comprehensive and is capable of reflecting the real needs of users. Hence Grounded Theory is selected as the research methods in this section.

### Grounded Theory

The Grounded Theory is established on the basis of empirical data, that is it derived from summarizing the original [data is continuously concentrated from bottom to top, conceptualized, categorized, and then systematically explored (Song et al., 2020)]. It can be seen that it can better identify the real semantic expression of users than cluster analysis. Therefore, in order to fully mine user needs and use the original data information, this paper utilizes Grounded Theory to mine user needs from online reviews.

Due to the large amount of comment data, it is necessary to sample the data before coding, extract representative sample data, and then study it based on Grounded Theory. Although the information obtained in this paper is mostly second-hand, the content, accuracy, and timeliness of the information are not much different from those of the first-hand because it keeps the information integrity. On the basis of ensuring the completeness and accuracy of the acquired data, the order of the data is shuffled, dispersed, and crushed, and the cleaned user online comments are analyzed sentence by sentence around the framework of the research theme. We use the stratified sampling method to sample the data because the amount of user online comment data after cleaning is very large, and the amount of comment data in each period varies. In order to ensures the representativeness of the samples with small sampling errors, and the comment data of each period is extracted. Generally, stratified sampling is also called type sampling, belonging to the branch of mathematical statistics, which refers to such a sampling method that samples (individuals) are randomly selected from different populations (layers) in accordance with a prescribed ratio from an object. The object can be divided into multiple different populations (layers), and samples drawn in each layer are independent of each other.

Before stratified sampling, the sample size should be determined firstly. Here, the Design Effect (*deff*) is used to determine the sample size. For stratified random sampling, the design effect is usually <1, which reflects the degree of decrease in the variance of the estimator. In order to ensure that the variance is small enough, the design effect of stratified sampling is taken as 1 (Luo, 2017). Since stratified sampling is a complex sampling, we determine the sample size *n*_*s*_ required for simple random sampling should be figured out firstly. The calculation formula is shown as follows:


(1) $$\begin{array}{l} n_{s}=Z_{\alpha /2}^{2}\times P\times \left(1-P\right)/\Delta^{2} \end{array}$$

Then according to the design effect *deff* of stratified sampling, the sample size *n* required for stratified sampling is calculated. The calculation formula is as follows:


(2) $$\begin{array}{l} n=n_{s}\times deff \end{array}$$

Since our data contains 47 WeChat APP update versions, it is regarded as a population composed of 47 layers in the sampling work. With a confidence level of 99% (1-α), the absolute error Δ is taken as 2%, and the overall proportion *P* is taken as 50% (at this time the sample size is the maximum value), the sample size of stratified sampling is 4,147. After the sample size is determined, the proportional distribution method in stratified sampling is selected to determine the sample number of each layer, i.e., the ratio of the sample number of each layer to the total number of the layer is equal. The specific sampling ratio and number of samples of the partial period are shown in Supplementary Material. After the collection and sorting of the original data are completed, the coding of the data samples is mainly divided into three steps, namely: open coding, axial coding, and selective coding.

#### Open Coding

Open coding is the first step to establish a theoretical system from a large amount of data based on Grounded Theory, requiring researchers to be highly sensitive to the theoretical system. As such, we deepen our understanding of the field by studying a lot of theoretical information about mobile APP products. Open coding mainly refers to the semantic analysis of sample data (a total of 4,147 comment data, and 100 comment data randomly selected for subsequent saturation testing) sentence by sentence, conceptualizing it, and merging overlapping concepts. Therefore, with the assistance of Nvivo11software, the initial coding of the comment data is manually carried out and the initial concepts are generated. The initial concepts generated are compared, and the intersected, similar or overlap concepts are merged, and 158 initial concepts are obtained. After removing the initial concepts that appeared <5 times, a total of 89 initial concepts were obtained. According to the semantic and connotative relationship between the initial concepts, the initial concepts are categorized and finally summarized into 15 initial categories. Since there are too many analysis sentences in open coding, only some representative ones are shown in Table 3.

**Table 3:** Partial open coding process.

**Original information (partial)**	**Initial conceptualization**	**Categorization**
Occupies too much memory and the start-up speed is slower and worse than before;	Large occupies	Memory optimization
Update quickly please; When will the update be pushed	Update is not timely Update notification is not timely	Update timeliness
Contacted customer service many times, directly through one	Failure to contact with after-sales	Response timeliness
Can't deal with the problem in time, no human customer service; Can't complain, customer service can't find	No customer service Customer service response is not timely	
Want to see moments' visitors;	Add Group visitors function	Functional insufficiency
Hope to add the function that allows to modify sent moments;	Add Group edit function	
Beauty function of moments please;	Add Beauty function	
It would be nice if I could change my WeChat ID	Modify WeChat ID	
Bad;	Bad experience	Subject experience
Good;	Good experience	
I personally feel QQ is good	Better user experience of competitive products	
Emoji icon is too big;	Large emoji	Functional insufficiency
There's a handling charge for cash withdrawals. It's rubbish	Fee for withdrawal	
Chat text background color cannot be modified;	Change word color	
Please cancel the rule that a bank card is required for real-name authentication;	cancel the rule that a bank card is required for real-name authentication	
Please save images over seven days	Prolong the duration of the chat history	
More beautiful after the update;	Better interface	Interface beauty
Simple, better and more useful;	Simple and good-looking interface	
Can you update some nice interface skin	Simple interface	
Suggest concise version	Function is not concise	
No dark mode	Dark mode	Interface friendliness
Chat history cannot be backed up automatically;	Back up chat history automatically	Functionality friendliness
Change the WeChat IDonce a month	Add ID modification frequency	
Repeated sound during video chat;	Unstable video chat	Functionality stability
Voice chat always interrupted;	Interrupted voice chat	
Cannot open the pushed information;	Unable to receive information	
Failure to restart video chat	Caton video chat	
Crash	Serious Crash	System stability
Annoying advertisement in moments	Too many advertisements	Advertising interruption
Cannot use card when forget password if not binding bank card;	Bank card binding	Account safety
Good for convenient communication and privacy protection;	Privacy protection	
Real-name authentication is required to receive red envelopes	Real-name authentication	
Good IOS system	Fluent operation of IOS system	Different requirements of different operating systems
Android memory is not large enough	Small Android memory	
When to update WeChat APP in Android system	Android update is not timely	

#### Axial Coding

Axial coding refers to the formation of the main category after the analysis and induction of the initial category obtained by the open coding. The 15 initial categories obtained by open coding are analyzed and summarized here before according to the definition of user needs in software engineering (Wang et al., 2019): (1) The conditions or capabilities required by the user to solve problems or achieve goals; (2) The capabilities of system and its component to meet the requirement from contracts, standards, specifications or other formal documents; (3) A document description that reflects the conditions or capabilities described in the above two scenarios, which mainly includes functional requirements and non-functional requirements. According to various needs of WeChat in different operating systems, we get a theoretical system composed of 6 main categories, namely 6 user needs of WeChat APP users obtained by Grounded Theory, which are: performance, reliability, usability, functional deficiency, functional insufficiency and system adaptability. Table 4 shows the correspondence between the main categories and the corresponding initial categories.

**Table 4:** Axial coding process.

**Main categories**	**Sub-categories**	**Introduction**
Performance	Memory optimization	User feedbacks on WeChat memory usage and installation package size
	Update timeliness	Timely push, internal testing, update cycle duration of WeChat version
	Feedback timeliness	Work efficiency of WeChat customer service, resolution of user complaints, etc.
Reliability	Subjective experience	The user's most direct experience of using WeChat
	Account safety	Involving WeChat payment, user privacy, account safety, etc.
	Advertising interruption	There are many advertisements on the chat interface and Moments of friends
	Interface friendliness	Dark mode, eye protection mode, etc.
	Interface aesthetics	Theme style diversity, background, question color settings
	Clear and concise functionality	The interface is clear, concise and not cumbersome
Availability	Functionality stability	The stability of the various functionalities of WeChat, such as flashbacks, caton, repeated voices, and echoes, etc.
	System stability	the overall system experience of the WeChat, the specific performance is whether the operation and interface are smooth
System adaptability	Different requirements in different operating systems	Due to the difference of operating system, the user experience is different, which in turn causes the user needs to be different
Functional deficiency	Functionalities completeness	Functionalities that users want to add
Functional insufficiency	Functionality friendliness	More user-friendly and easy-to-use functionalities for users
	Functionality optimization	Functions that users want to improve

#### Selective Coding

In order to study the impact of user needs on APP downloads, user comments are coded to form initial concepts and main categories, and then further integrated and condensed. By sorting out the logical relationship between categories, it is found out that all six categories are closely related with the volume of WeChat downloads.

#### Saturation Test of Grounded Theory

Saturation test of Grounded Theory refers to the completeness test of the user needs theoretical system formed during its three coding processes. Here, a theoretical saturation test is performed on 100 comment data which was previously reserved. Focused on the core category of WeChat APP downloads, there is no new initial concepts, new categories, or structural relationships appeared in the coding process. Therefore, it is determined that the main category and initial concept are relatively complete, and the theoretical model obtained here has reached the saturation state through the theoretical saturation test.

## Construct the Model

Online comments not only contain users' emotional attitude, satisfaction, rating and other factors toward products, but also user needs information as can be seen from Data Acquisition and Preprocessing (or Preparing). When users download the APP, they will take these factors into consideration through online comments and then decide whether to download the APP. Previous research reveals that sentimental orientation of reviews (e.g., positive, neutral, negative), degrees of satisfaction and star ratings have influences on APP downloads as well (Hao, 2010; Ju, 2017). So as for the second question- how online reviews affect downloads, this section manages to explain how online reviews influence downloads by studying the relationship between these factors and the volume of APP downloads. In this section, we focus on finding the suitable model to establish the relationship between download volume and the above factors and answer the second question based on the results.

### Model Selection

Regression models are widely adopted to analyze the causal or quantitive relationship between among multiple variables. Traditional regression models consist of logistic regression, stepwise regression, ridge regression, etc. Yet they mainly focus on mathematical expectations of explanatory variables and come with strict hypothetical conditions. In addition, the process of backward stepwise regression to gradually eliminate variables is irreversible. In order to optimize the model, some non-statistically significant explanatory variables may be retained. If someone pays attention to the relationship between the median of the explained variable and other quantiles and the explanatory variable, quantile regression is a very good choice. Compared with the traditional simple regression model, quantile regression has multiple advantages. Firstly, from the perspective of the research scope, quantile regression is capable to describe the whole picture of the research object in a more comprehensive manner. Secondly, in terms of condition assumptions, general linear regression needs to meet a series of strong assumptions (independence, normality, homoscedasticity), which is often impractical in reality. In contrast, the condition assumptions of quantile regression are much weaker (independence). From the perspective of outlier influence, quantile regression estimator is not susceptible to outlier influence, so the estimator is more robust (Sun, 2019).

The quantile regression model was first proposed by Koenker and Bassett (1978). They generalized the Least Absolute deviation regression and developed the quantile regression model, which was used to estimate the conditional quantile function of a given independent variable. Quantile regression is a semi-parametric technique widely used in economics. Currently, quantile regression model has been widely used in many fields due to its good properties (Chen et al., 2022b,c). For example, Buchinsky (1994) applied quantile regression to the study of the change of wage structure in the United States, and the results under different quantiles reflected the change of wage inequality. Liu and Deng (2021) used STIRPAT model of fixed effects panel quantile to test the impact of per capita GDP, population size, energy intensity, fixed wage investment and “Ten policies” on carbon emissions at different quantile levels. Here we introduce the quantile regression model to build up the relationship between quantile of APP downloads and user needs,review emotional tendency, user satisfaction and star ratings.

### Quantile Regression Analysis

#### Research Variables and Measurements

This section analyzes the emotional tendency of users in online reviews through emotional analysis of online reviews during which Likert method was used to quantify user satisfaction. Meanwhile, user needs are quantified as follows. This paper analyzes the word frequency of the sample comment data based on 6 user needs obtained in the previous section. It screens out 100 of the most frequently used keywords of each user needs factor and establishes auxiliary words group to serve as the representative vocabulary of each factor. At the same time, according to the auxiliary words group of each factor, a regular expression is established to filter out reviews that have intersections with auxiliary word group from the overall. In addition, for six user needs, proportions of them in total comments are calculated. The proportion can represent the degree of user attention of the influencing factor with respect to the corresponding attribute in a certain period. Obviously, the higher the proportion is, the higher the user's attention is paid to the attribute and the more important this factor will be. Therefore, those proportions can be used as the quantitative value of the factor in each period. The increase of WeChat downloads during each period from Qimai Data Platform (https://www.qimai.cn) is regarded as the APP downloads. Since the download volume of the WeChat APP is very large in each period, the logarithmic function is performed to the APP download volume to lower the download scaling length in order to ensure more stable data without changing the nature and correlation of it.

The variables involved in the model are shown in Table 5 (hereinafter referred to as user needs, ratings, user satisfaction, and emotion tendencies as explanatory variables).

**Table 5:** Variables description.

**Variables**	**Symbols**	**Description**	**Measuring way**
LN (downloads)	LNY	The logarithm of WeChat's downloads in each cycle	LN (Number of APP downloads)
Percentage of negative emotions	neg	The snownlp package in python is used to analyze the emotion of users' online comments, and the positive and negative emotion express users' emotion tendency toward WeChat. Since neutral emotion has little effect on user downloading behavior, the proportion of positive emotion and negative emotion is selected as two explanatory variables. Among them, the sum of the three proportions (positive, negative, neutral) is 1.	Positive (negative) comments for each version/total comments for each version
Percentage of positive emotions	pos		
Proportion of general satisfaction	*X*_1_	The user's satisfaction with the use of the WeChat APP is divided into 6 based on Likert's score: Highly satisfied>moderately satisfied>generally satisfied>generally dissatisfied>moderately dissatisfied>highly dissatisfied, and $\sum_{i=1}^{6}X_{i}=1$	ROSTCM6 was used to conduct Likert rating on user comments periodically. Likert rating divided the emotion of each comment into six sections, and the score obtained was regarded as user satisfaction. They were highly satisfied (20 points and above 20 points), moderately satisfied (10–20 points), generally satisfied (0–10 points), highly dissatisfied (−20 points and below 20 points), moderately dissatisfied (−20~ (−10 points) and generally dissatisfied (−10~0 points). The indicator is calculated as follows: number of comments in each category/Total number of comments
Proportion of middle satisfaction	*X*_2_		
Proportion of high satisfaction	*X*_3_		
Proportion of general dissatisfaction	*X*_4_		
Proportion of middle is satisfaction	*X*_5_		
Proportion of highdissatisfaction	*X*_6_		
One-star ratio	*S*_1_	The user's rating of the WeChat APP experience is divided into 1~5 stars. The higher the star rating is, the better the user experience will be, and it satisfies$\overset{5}{\underset{i=1}{\sum }}S_{i}=1$	number of reviews per star/total reviews
Two-star ratio	*S*_2_		
Three-star ratio	*S*_3_		
Four-star ratio	*S*_4_		
Five-star ratio	*S*_5_		
Functional deficiency	*F*_1_	User needs to add new functionalities	Word segmentation and word frequency analysis were carried out on all comment data. Keywords with top 100 frequency were selected to form the representative lexicon of each need. Regular expressions are built in Python software to classify comments into the corresponding requirements based on keywords. We take the proportion of the number of user reviews for each requirement category in the total effective reviews of the cycle as the user attention for each requirement in the cycle.
Functional insufficiency	*F*_2_	User needs to improve WeChat's existing functionalities	
Performance needs	*pro*	Compliance with timeliness and resource economy requirements	
Availability needs	*ava*	Probability of operation without failure in a certain period of time	
Reliability needs	*rel*	The degree to which users are less mistaken and satisfactory, that is, the user's subjective perception of the software	
Different requirements of different operating systems	*syd*	Due to different operating systems, users have different experience in using WeChat, which in turn leads to different user needs	

#### Model Construction

On the basis of quantifying user needs, the construction of a regression model requires observing the probability distribution of each variable to test whether it meets the model's assumptions (normality assumption). Through statistical analysis, it is found that there is a skew distribution in the proportion of downloads (Normal probability graph of downloads is shown in Supplementary Material), ratings, and emotional tendencies. And we had knew the traditional multiple linear regression model is no longer suitable for this manner of data. Yet the quantile regression model does not require the normality of the data.

The model is as follows:


(3) $$\begin{array}{l} Q_{\tau }\;(y_{i})=\beta_{0}\;(\tau )+\beta_{1}\;(\tau )\;x_{i1}+\beta_{2}\;(\tau )\;x_{i2}+\cdots +\beta_{p}\;(\tau )\;x_{p2}\\ \;+\varepsilon \;(\tau )\;i=1,2,\cdots \;,n \end{array}$$

where τ is the quantile point, β_*i*_ is no longer a constant but a function of the quantile, and ε (τ) is the error. The minimized objective function is:


(4) $$\begin{array}{l} \overset{n}{\underset{i=1}{\sum }}\rho_{\tau }\;(y_{i}-x_{i}^{\prime }\beta )\;i=1,2,\cdots \;,n \end{array}$$

Loss function is defined as follows:


(5) $$\begin{array}{l} \rho_{\tau }\;(\varepsilon )=\varepsilon \;(\tau -I\;(\varepsilon )) \end{array}$$

(6) $$\begin{array}{l} I\;(\varepsilon )=\{\begin{array}{c} 0,\varepsilon \geq 0\\ 1,\varepsilon <0 \end{array} \end{array}$$

where $x_{i}={\left(1,\;x_{i1},\;x_{i2},\cdots \;,\;x_{p2}\right)}^{\prime },\;i=1,2,\;\cdots \;,n$.

With regarding to the construction of the quantile regression model, our study selects the quantile points of APP downloads 0.1, 0.15, 0.2, 0.25, 0.3, 0.35, 0.4, 0.45, 0.5, 0.55, 0.6, 0.65, 0.7, 0.75, 0.8, 0.85, 0.9, 0.95 to perform quantile regression. The quantile regression model is tested for equal slope and Wald test. The quantile regression coefficient Table and its test results are shown in Tables 6–8.

**Table 6:** Quantile regression coefficient significance table for each quantile (quantile 0.1 ~ 0.35).

**Variables**	***q***					
	**0.1**	**0.15**	**0.20**	**0.25**	**0.30**	**0.35**
*intercept*	-	-	1.21E-9	0.003	0.015	-
*pos*	-	-	-	-	0.012	0.040
*neg*	-	-	-	-	-	-
*X*_1_	-	-	0.000023	-	-	-
*X*_2_	-	-	0.000015	-	-	-
*X*_3_	-	-	0.000010	-	-	-
*X*_4_	-	-	0.02	0.03	0.01	-
*X*_5_	-	-	0.000005	-	-	-
*X*_6_	-	-	1.058E-8	0.000385	0.000253	0.002
*S*_1_	-	-	6.09E-8	6.48E-7	2.01E-8	0.000006
*S*_2_	-	-	0.000062	0.006	0.001	0.011
*S*_3_	-	-	-	-	-	-
*S*_4_	-	-	7.23E-7	-	-	-
*S*_5_	-	-	-	-	-	-
*F*_1_	-	-	0.001	-	-	-
*F*_2_	-	-	0.000005	0.011	0.012	-
*pro*	-	-	0.000010	-	-	-
*ava*	-	-	-	-	0.007	-
*rel*	-	-	-	-	-	-
*syd*	-	-	0.000246	-	-	-

**Table 7:** Quantile regression coefficient significance Table for each quantile (quantile 0.40 ~ 0.65).

**Variables**	***q***					
	**0.40**	**0.25**	**0.50**	**0.55**	**0.60**	**0.65**
*intercept*	-	-	-	-	-	-
*pos*	-	-	-	-	0.018	0.004
*neg*	-	-	-	-	-	-
*X*_1_	-	-	-	-	-	-
*X*_2_	-	-	-	-	-	-
*X*_3_	-	-	0.049	0.026	0.025	0.009
*X*_4_	-	-	-	-	-	-
*X*_5_	-	-	-	-	-	-
*X*_6_	0.009	0.046	0.010	0.025	0.000006	9.376E-7
*S*_1_	0.000072	0.002	0.008	0.005	0.000441	0.000071
*S*_2_	-	-	-	-	-	-
*S*_3_	-	-	-	-	-	-
*S*_4_	-	-	-	0.043	0.014	0.002
*S*_5_	-	-	-	-	-	-
*F*_1_	-	-	0.047	0.017	-	-
*F*_2_	-	-	-	-	-	0.021
*pro*	-	-	-	-	-	-
*ava*	-	-	-	-	-	-
*rel*	-	-	-	-	-	-
*syd*	-	-	0.008	0.003	0.014	0.005

**Table 8:** Quantile regression coefficient significance Table for each quantile (quantile 0.70 ~ 0.95).

**Variables**	***q***					
	**0.70**	**0.75**	**0.80**	**0.85**	**0.90**	**0.95**
*intercept*	-	-	-	-	-	-
*pos*	0.002	0.000046	-	-	-	-
*neg*	-	0.047	-	-	-	-
*X*_1_	0.047	0.010	-	-	-	-
*X*_2_	0.046	0.007	0.000	-	-	-
*X*_3_	0.002	0.000182	-	-	-	-
*X*_4_	-	-	-	-	-	-
*X*_5_	-	0.009	-	-	-	-
*X*_6_	8.371E-7	1.92E-9	-	-	-	-
*S*_1_	0.000118	1.56E-7	-	-	-	-
*S*_2_	-	0.040	-	-	-	-
*S*_3_	-	-	-	-	-	-
*S*_4_	0.000033	0.00003	-	-	-	-
*S*_5_	-	-	-	-	-	-
*F*_1_	0.031	-	-	-	-	-
*F*_2_	0.047	0.001	-	-	-	-
*pro*	-	0.022	-	-	-	-
*ava*	-	-	0.001	-	-	-
*rel*	-	-	-	-	-	-
*syd*	0.000032	2.37E-7	0.00002	-	-	-

*The values in the Table are the significant variables under the quantile, and the blank value indicates that the variable is not significant under the quantile*.

#### Model Testing

Generally speaking, there are two types of tests for quantile regression models, namely model testing and serial quantile regression testing. Model testing includes goodness of fit test, quasi-likelihood ratio test, and Wald test. Serial quantile regression tests include slope equality test, symmetry test, etc. This paper performs quasi-likelihood ratio test, the goodness of fit test, Wald test are shown and slope equality test respectively on quantile regression models. The results and detailed description of the quasi-likelihood ratio test, the goodness of fit test, Wald test and slope equality test are shown in Supplementary Material. According to the results, the models at 0.2, 0.25, 0.3, 0.35, 0.5, 0.55, 0.6, 0.65, 0.7, 0.75, 0.8 quantiles have good fitting effect with its explanatory variables being highly valid.

Regarding the slope equality test, here is the analysis around the estimated parameter graph in the SPSS output result. Usually, the slope equality test is whether the estimated structural parameters (slope) are equal for different quantiles. The estimated parameter map demonstrates that at different quantiles, the estimated coefficients of most variables have relatively large changes. That is, at quantiles with different downloads, once the influence of the explanatory variables is different, the model can be considered to pass the slope equality test and the series quantile regression test.

#### Analysis of Regression Model Results

From the quantile regression results (Tables 6–8), it can be seen that no matter which quantile it is, the proportion of three-star rating (*S*_3_), the proportion of five-star rating (*S*_5_), and reliability (rel) are different. Significantly, it can be seen that these two explanatory variables have almost no effect on APP downloads. Highly dissatisfied (*X*_6_), the proportion of one-star rating (*S*_1_), functional insufficiency (*F*_2_), and the differential requirements of different systems (*syd*) have significant impact on almost all quantiles. Here, the 0.1–0.4 quantile is recorded as the low download stage, 0.4–0.65 is recorded as the medium download stage, and 0.65–0.95 is recorded as the high download stage. The detailed analysis results are as follows:

##### Functionality Needs

**Figure 2:**
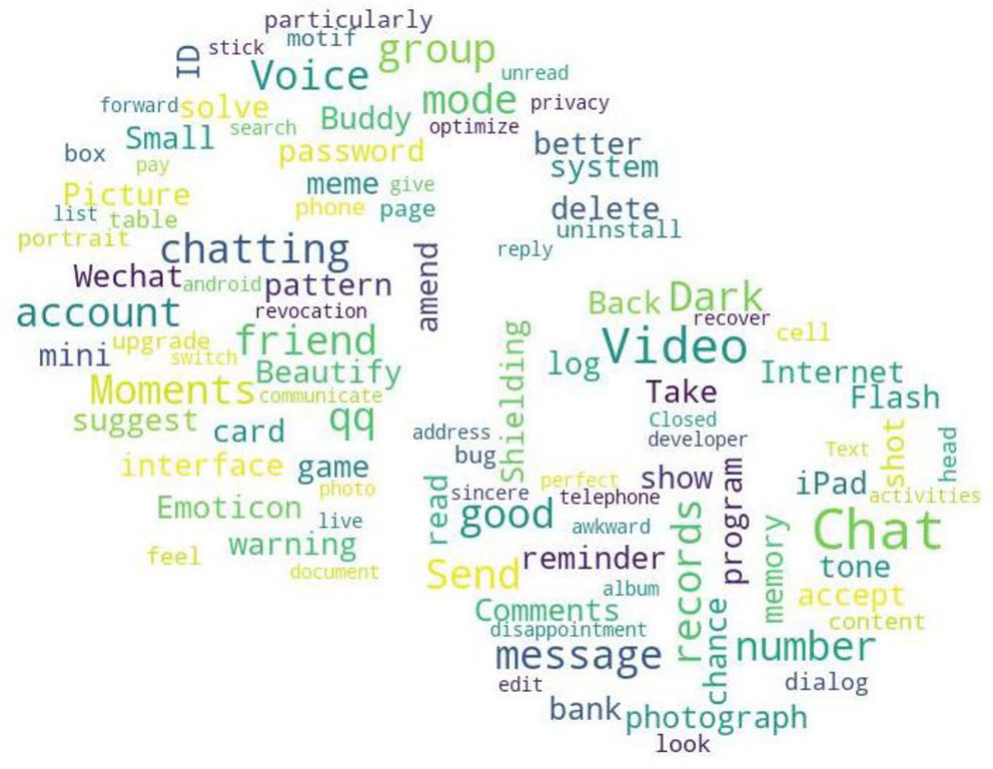
Functions deficiency on WeChat.

**Figure 3:**
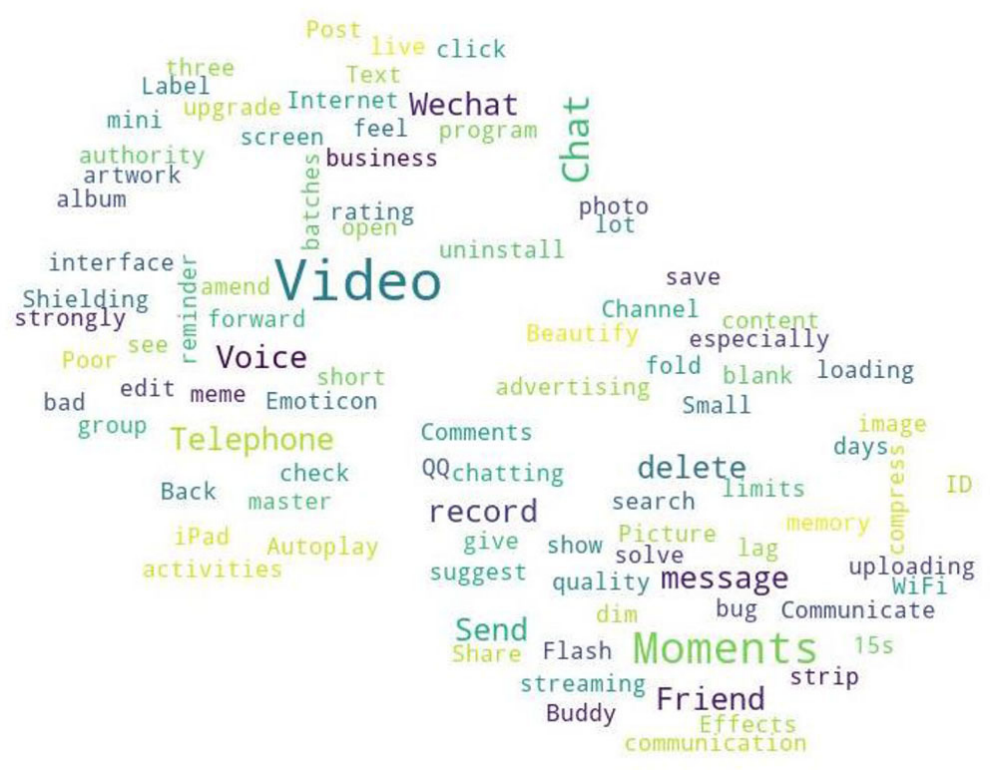
Functional insufficiency in WeChat.

From the quantile regression coefficient significance Tables 6–8, it can be seen that *F*_1_ and *F*_2_ are more significant in the medium and low download stages. Compared to *F*_1_, *F*_2_ has more significant quantile points, meaning when the WeChat APP download volume is not large, the attention of user needs is mainly focused on the functional deficiency and improved. Compared to the development of new functional modules, WeChat developers should pay more attention to the improvement of existing functionalities. This paper screens out the needs to be added and to be improved for WeChat APP in online reviews according to the auxiliary words group and conducts word frequency analysis to obtain the top 100 keywords in word frequency rankings, and draws a word cloud diagram, including needs for features to be added and to be improved. The required word cloud diagrams are shown in Figures 2, 3. The larger font size in the word cloud diagram indicates there are more users who have needs for the functionality. From Figure 2 and based on the original data of user comments, we can see that modifying the WeChat ID or other functionalities related to the WeChat ID, video beautifying functionality, chat grouping, dark mode and other functionalities are urgently needed by users and important for WeChat companies. As can be seen from Figure 3, with regarding to the improved functionalities of the WeChat APP, combined with the original online comments, it is not difficult to find that the users' main concerns are WeChat ID modification, Moments, chat (grouping), record retention time, beautify, etc.

##### Performance, Availability, Reliability

**Figure 4:**
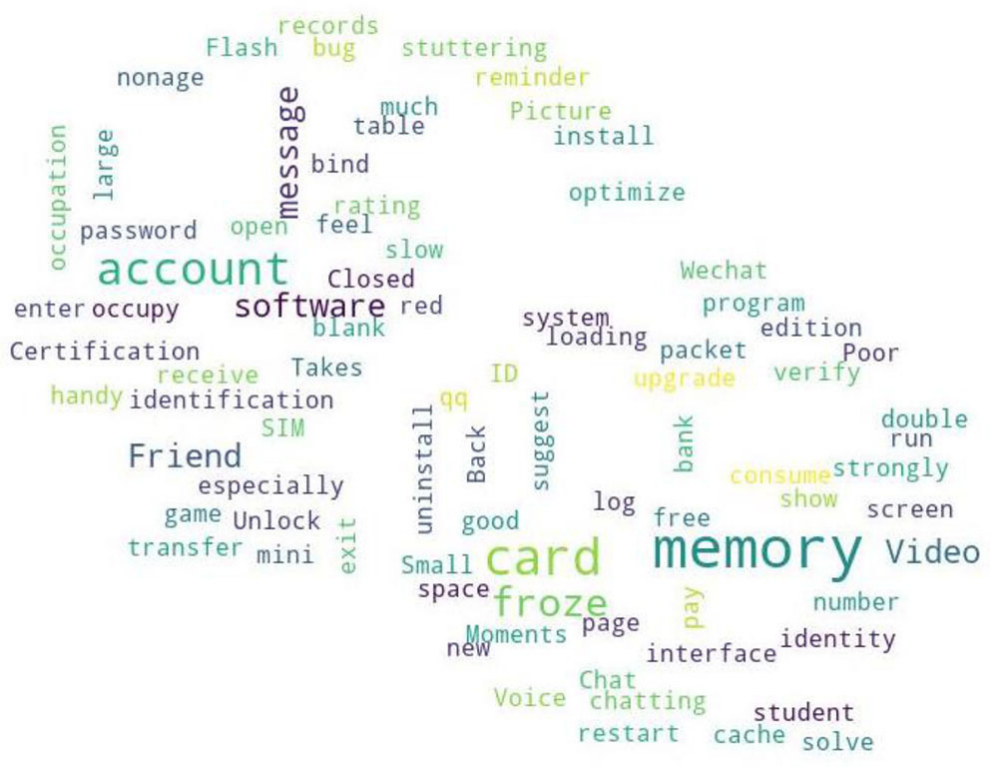
Function needs of WeChat.

**Figure 5:**
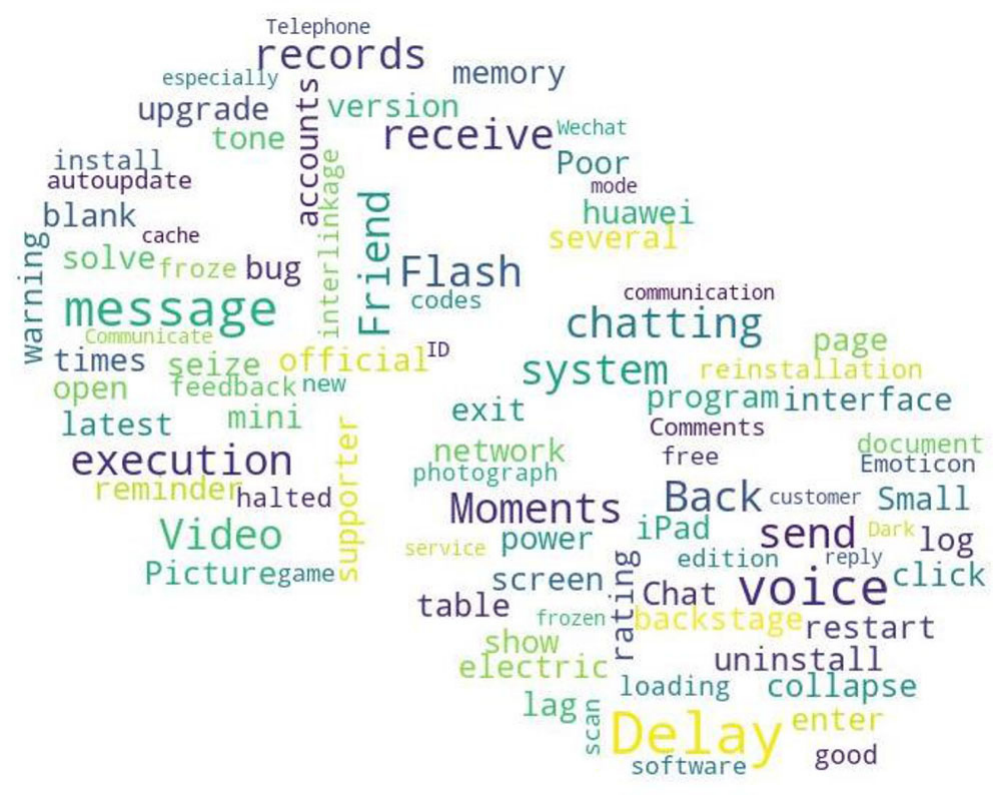
Availability of WeChat.

From the quantile regression coefficient significance Tables 6–8, it can be seen that *rel* has no significant impact on any quantile, *pro* only significantly impacts on the quantiles 0.2 and 0.75, and *ava* only significantly impacts on 0.3 and 0.8. In the medium download stage, users have no requirements for the non-functional requirements of the WeChat APP, while in the low or high download stage, WeChat developers need to pay attention to the performance requirements and usability requirements of the APP. Comparing the results of the functional requirements analysis, it is found that the user needs attention is mainly focused on the functional requirements category. Since *pro* and *ava* have a significant impact on the WeChat APP downloads in the low and high stages, a word frequency analysis of the user comments is conducted, and the results are shown in Figures 4, 5. Figure 4 shows that the user's focus on WeChat performance is froze, crash and memory. Figure 5 shows that the user's focus on the usability of WeChat is unable to open, delay, blank screen, flash back, etc., and these phenomena often appear in WeChat Moments and video functions.

##### Different Needs of Different Operating Systems

**Figure 6:**
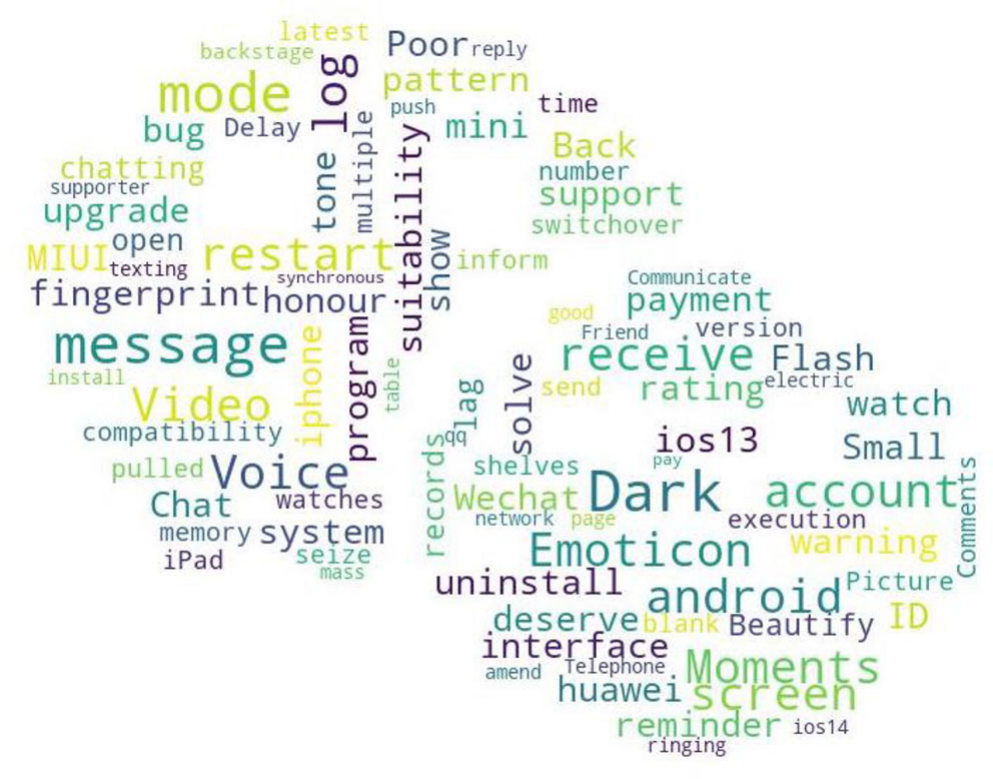
Different requirements of WeChat on different operating system.

Before the release of Huawei Harmony Operation system, there are mainly two mobile phone operating systems on the market: IOS and Android. Generally, users of different operating systems have different experiences while using WeChat APP. In addition, due to different systems, there will be slight differences in the update time, functionalities, and operation interface of the WeChat APP. From the quantile regression coefficient significance Tables 6–8, it can be seen that the different needs in different operating systems have a significant impact on the medium or high downloads stage. It can be seen from parameter estimates that the linear model coefficient of the different needs variables of different systems at this time is <0, indicating that it has a negative impact on the downloads. Generally speaking, different experiences will cause users to have a comparative psychology to some of the functionalities of WeChat APP, thereby forming negative emotions, which affect the download behavior of WeChat users. At this time, the negative emotions of users brought about by the different needs of different systems are often an aspect that enterprise developers tend to ignore. Here, the negative comments brought about by the different needs of different systems are screened out, word frequency analysis is performed, and a word cloud diagram is drawn. The result is shown in Figure 6, which indicates that the differences in user needs of WeChat APP due to different systems mainly result from the difference in background mode (dark mode), the difference in experience of crashing, and the difference in the functionality of modifying the WeChat ID.

##### Emotion Tendency

Neutral emotional comments generally have no effect on APP downloads, so this paper only studies the impact of the proportion of users' positive and negative emotions on downloads. From the quantile regression coefficient significance Tables 6–8, it can be seen that in the low and medium download stages, *pos* is significant, and *neg* only affects the 0.75 quantile of downloads. It can be seen from parameter estimates that when the number of WeChat APP downloads is not high, the coefficient of *pos* in the linear model is >0, so it is believed that the positive emotions of users' online comments can promote downloads. At this time, WeChat developers should pay attention to users' negative emotional comments and combine the above-mentioned research on features to be added, features to be improved, usability and performance, analyze the reasons for the formation of negative emotion, and make improvements.

##### Ratings

The rating represents the user's overall evaluation of the user experience of WeChat APP. From the quantile regression coefficient significance Tables 6–8, it can be seen that *S*_3_ and *S*_5_ are not significant for any quantile of downloads. In the low download stage, the influence of *S*_1_ and *S*_2_ is significant. In the medium download stage, the influence of *S*_1_ and *S*_4_ is significant. Therefore, if WeChat developers want to increase APP downloads, they should focus on one-star user reviews, which is also in line with reality. The reason is that a one-star rating means users have the worst experience in using WeChat, and they tend to avoid APPs with low star ratings on their own.

##### User Satisfaction

User satisfaction represents the degree of user satisfaction with the WeChat APP. From the quantile regression coefficient significance Tables 6–8, it can be seen that in the low download stage,*X*_1_, *X*_2_, *X*_3_, *X*_4_, *X*_5_, and *X*_6_ all have a significant impact. In the medium download stage, only*X*_3_, *X*_4_ and *X*_6_ have a significant impact. In the high download stage, only *X*_6_ has a significant impact. It can be seen that high dissatisfaction (*X*_6_) has a significant impact on WeChat APP downloads at any stage.

## Conclusions

### Research Work and Conclusions

This paper uses WeChat APP as the research object. Based on Grounded Theory, it digs out user needs from online user comments, and builds a stepwise regression model and quantile regression model for users' emotion tendency, user satisfaction, users' star ratings, and the degree to which users are concerned about requirements (word frequency analysis, sentiment analysis are used to quantify user needs) and APP downloads. The analysis results show that Quantile regression model can better explain the impact of these variables on downloads.

In addition, by mining user needs and studying the relationship between online reviews and APP downloads, the following conclusions are drawn:

1. User needs based on online comment mining include performance, reliability, availability, differentiated requirements of different systems, functional deficiency, and functional insufficiency. The main requirements of users in each part are shown in Supplementary Material.
2. Reliability, three-star rating and five-star rating have no significant impact on APP downloads. High dissatisfaction, one-star rating, functional insufficiency, and differentiated requirements of different operating systems have an impact on the download volume of almost any quantile.
3. When considering the functionality requirements of users, companies should pay attention to the improvement of existing functionalities. At the same time, for the improvement of existing functions, companies need to focus on WeChat ID modification, Moments, chat (group), and the retention time of chat records and other functionalities. For functional deficiency, companies should focus on other functionalities of WeChat, video beautify, chat grouping, dark mode, WeChat multiple login (mobile phone, Tablet, computer) and other functionalities.
4. For non-functionality requirements, companies should focus their attention on the performance requirements, availability requirements, and system adaptability. Among them, the user's performance requirements are mainly to solve the phenomenon of stalls, flashbacks, and memory. The user's usability requirements are mainly to solve the phenomenon of inability to open, delay, black screen, and flashback. The different needs of different systems are mainly background mode (dark mode), the experience difference of the crash due to the different requirements of systems and the difference in the function of modifying the WeChat ID, etc.

### Research Limitations and Future Research

Due to the influence of artificial error or other factors on the research work, the following assumptions are made in this paper:

1. There may be some errors in data cleaning: invalid comments (blank lines, advertisements and comments unrelated to products) may not be completely removed, so it is assumed that all invalid comments have been removed;
2. There may be some errors caused by manual coding during the Grounded Theory coding, so it is assumed that there is no error in all coding;
3. The premise of quantile regression model is the independence of data, which assumes that various user needs are independent of each other.

In addition, this paper still has the following shortcomings which need further study.

1. As WeChat is popular in other countries, attention should be paid to user needs in those regions as well. This paper only studies the needs of Chinese users. Therefore, in the follow-up research, it is necessary to further analyze the application and promotion of WeChat APP in other countries.
2. Although this paper analyzes the focus of user needs, different user groups have different needs. Further research is expected to divide up users to groups according to their characteristics, needs, and preferences in order to provide them more specific services (Chen et al., 2022a).
3. This paper can dynamically and accurately extract user needs, so it can effectively guide the enterprise's software update strategy, and has important application prospects; however, it is still in the stage of theoretical research, and will cooperate with enterprises in the future to apply relevant methods to enterprise practice.

## Data Availability Statement

The data used to support the findings of this study are available from the corresponding author upon request.

## Author Contributions

TC described the proposed framework and wrote the whole manuscript. CZ implemented the simulation experiments. JY and GC collected data and revised the manuscript. All authors have read and agreed to the published version of the manuscript.

## Funding

This research is supported by the National Social Science Foundation of China (Grant No. 20BTQ059), the Zhejiang Provincial Natural Science Foundation of China (Grant No. LY22G010003), the Project of China (Hangzhou) Cross-border E-commerce College (Grant No. 2021KXYJ07), the Contemporary Business and Trade Research Center and Center for Collaborative Innovation Studies of Modern Business of Zhejiang Gongshang University of China (Grant No. 14SMXY05YB), Hubei Key Laboratory of Mechanical Transmission and Manufacturing Engineering (MTMEOF2021A01), and the Characteristic & Preponderant Discipline of Key Construction Universities in Zhejiang Province (Zhejiang Gongshang University-Statistics), as well as Collaborative Innovation Center of Statistical Data Engineering Technology and Application.

## Conflict of Interest

The authors declare that the research was conducted in the absence of any commercial or financial relationships that could be construed as a potential conflict of interest.

## Publisher's Note

All claims expressed in this article are solely those of the authors and do not necessarily represent those of their affiliated organizations, or those of the publisher, the editors and the reviewers. Any product that may be evaluated in this article, or claim that may be made by its manufacturer, is not guaranteed or endorsed by the publisher.
