# Supplementary material for: Grounded Theory-Based User Needs Mining and Its Impact on APP Downloads: Exampled With WeChat APP
Source: Front Psychol. 2022 Jun 14;13:875310. doi: 10.3389/fpsyg.2022.875310 (PMC9237435; doi:10.3389/fpsyg.2022.875310)
Supplement: Supplementary file 1 [file Image_1.pdf]

Supplementary Material

1 Supplementary Figures

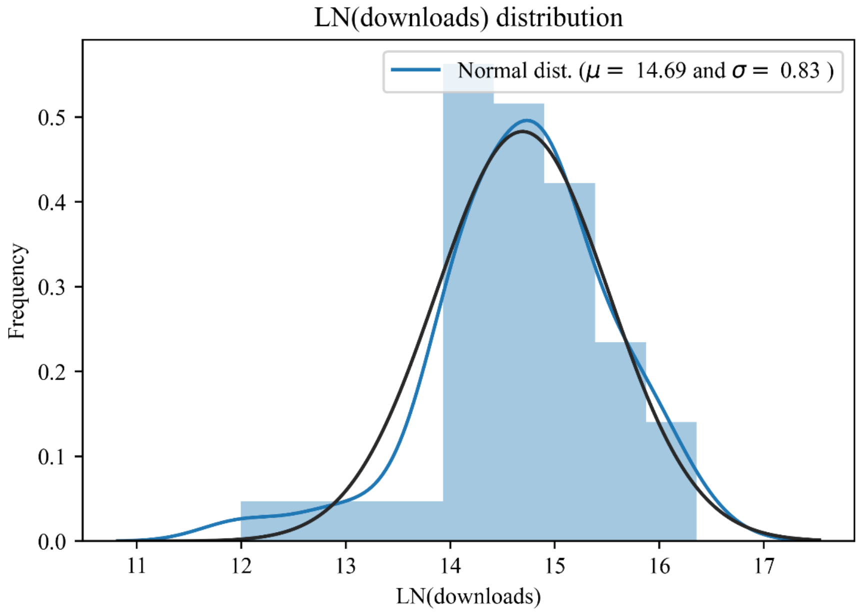

Figure 1 Normal probability of downloads

Supplementary Figures 1. The normal probability graph of downloads is shown in Figure 1, and the skewness and kurtosis of downloads are -0.746108 and 1.784425 respectively. It can be seen that the download is left-biased and the kurtosis of its distribution is steep.

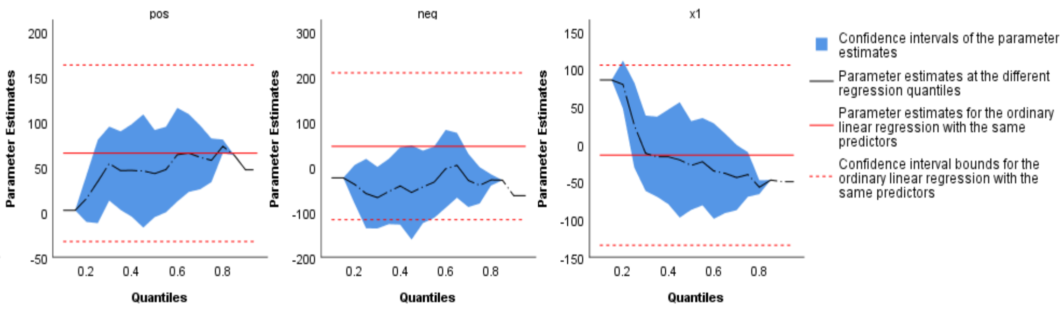

Figure 2 *Pos*, *neg*, $\boldsymbol{X_1}$ parameter estimates

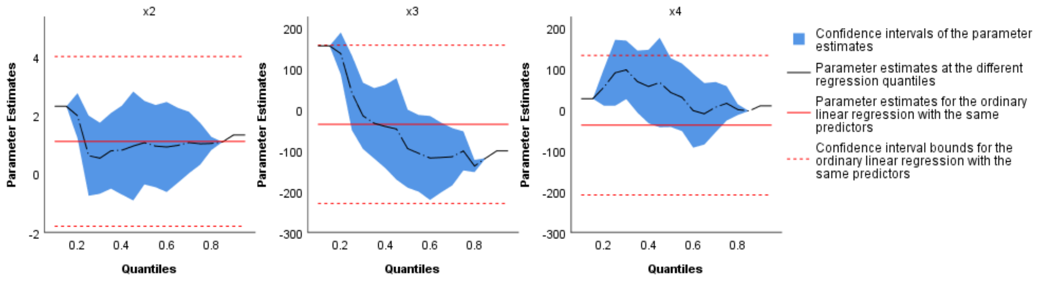

Figure 3 $\boldsymbol{X_2}$, $\boldsymbol{X_3}$, $\boldsymbol{X_4}$ parameter estimates

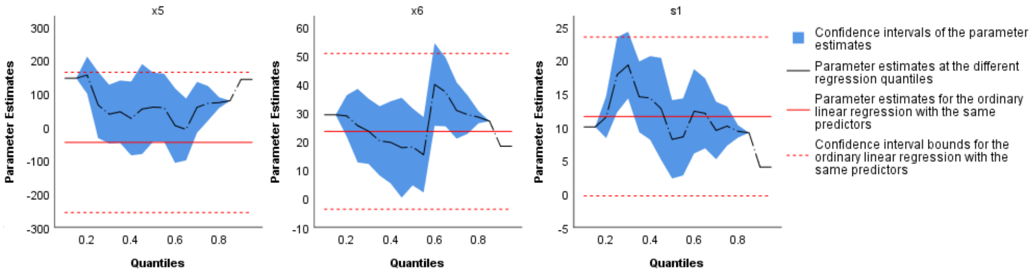

Figure 4 $\boldsymbol{X_5}$, $\boldsymbol{X_6}$, $\boldsymbol{S_1}$parameter estimates

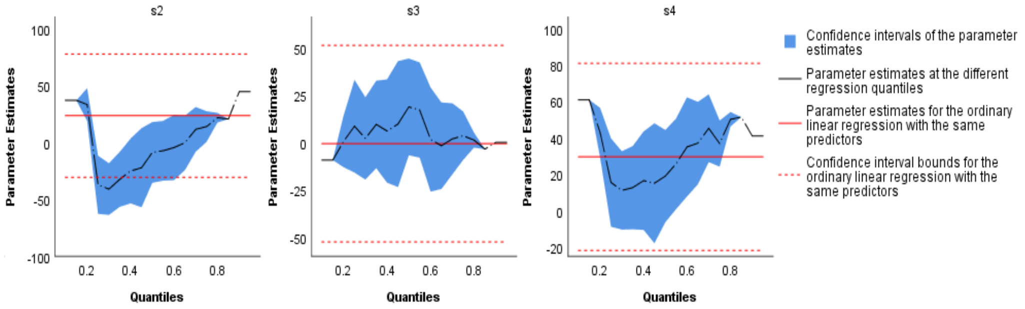

Figure 5 S_2, S_3, S_4 parameter estimates

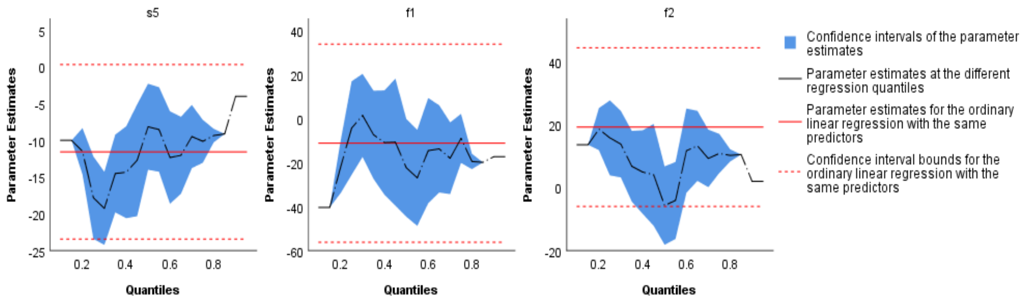

Figure 6 S_5, F_1, F_2parameter estimates

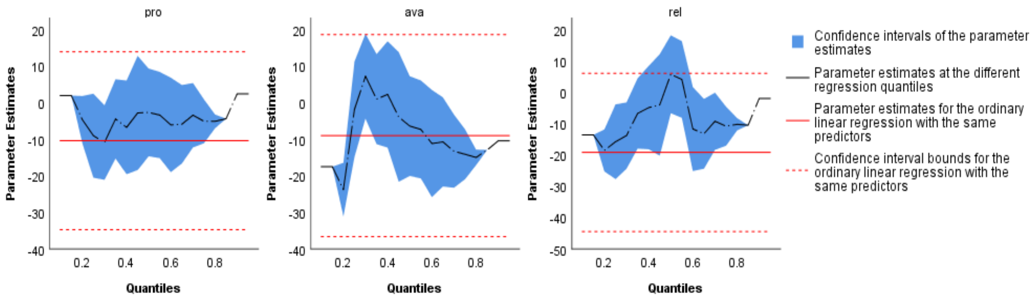

Figure 7 *Pro*, *ava*, *rel* parameter estimates

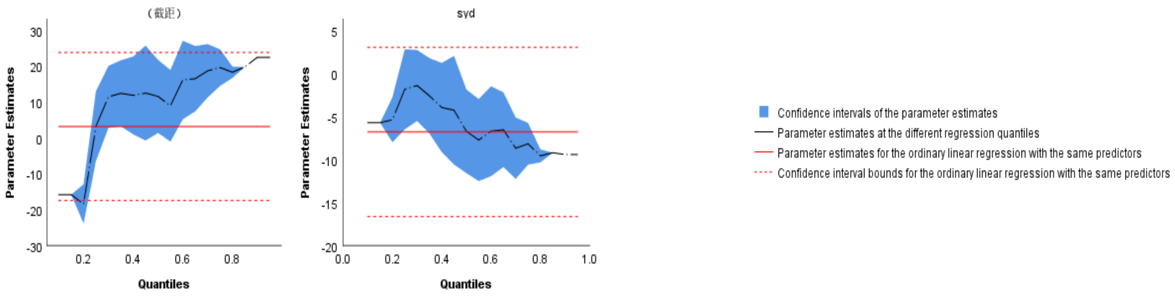

Figure 8 *Intercept*, *syd* parameter estimates

Supplementary Figures 2-8. The estimated parameter map (Figures 2-8) shows the structural parameters and their confidence intervals of the explanatory variables at different quantiles (abscissa) of APP downloads, as well as the structural parameters and their confidence intervals of general linear regression.
