# Supplementary material for: Grounded Theory-Based User Needs Mining and Its Impact on APP Downloads: Exampled With WeChat APP
Source: Front Psychol. 2022 Jun 14;13:875310. doi: 10.3389/fpsyg.2022.875310 (PMC9237435; doi:10.3389/fpsyg.2022.875310)
Supplement: Supplementary file 2 [file Table_1.pdf]

Supplementary Material

1 Supplementary Tables

Table 1 Partial periodic sampling detail

Version period	6503	6504	6506	···	802	803	806	807	809
Overall(piece)	17654	24888	4284	···	9222	21626	15193	21134	17950
Sample proportion	0.6%	0.6%	0.6%	···	0.6%	0.6%	0.6%	0.6%	0.6%
Sample (piece)	106	149	26	···	55	130	91	127	108

Supplementary Table 1. The specific sampling ratio and number of samples of the partial period are shown in Table 1.

Table 2 Quasi-likelihood ratio test result

Quantile *q*	Goodness of fit (0,1)	Quasi-LR statistic	Prob (Quasi-LRstat)
0.1	0.598	-	11.283022(>0.05)
0.15	0.449	-	1.203632(>0.05)
0.2	0.725	30.324	0.034412
0.25	0.704	667.591	0.000000
0.3	0.889	29.927	0.046836
0.35	0.790	29.890	0.045273
0.4	0.392	-	0.062030(>0.05)
0.45	0.874	-	0.050722(>0.05)
0.5	0.539	29.676	0.041924
0.55	0.648	31.384	0.032836
0.6	0.858	31.477	0.034829
0.65	0.775	33.464	0.014658
0.7	0.901	437.487	0.000000
0.75	0.633	29.654	0.040948
0.8	0.481	29.997	0.048826

0.85	0.521	-	0.728371(>0.05)
0.9	0.768	-	0.052830(>0.05)
0.95	0.511	-	0.329372(>0.05)

Table 3 The result of Wald test (q = 0.2, α=0.05)

Variable	Coefficient	Standard Error (S.E.)	Wald	Prob
intercept	-48.440	5.2726	84.404	0.000
pos	14.923	12.6297	1.396	0.248
neg	-38.773	21.0279	3.400	0.077
x1	79.638	15.4636	26.523	0.000
x2	1.990	0.3745	28.229	0.000
x3	136.683	24.9454	30.022	0.000
x4	54.514	21.9865	6.148	0.020
x5	154.986	27.0789	32.759	0.000
x6	28.845	3.5088	67.583	0.000
s1	11.474	1.5340	55.948	0.000
s2	33.365	6.9937	22.760	0.000
s3	0.663	6.6898	0.010	0.922
s4	42.978	6.6329	41.985	0.000
s5	0	-	-	-
f1	-21.903	5.8067	14.228	0.001
f2	18.579	3.2571	32.536	0.000
pro	13.927	2.5447	29.954	0.000
ava	-5.216	4.5472	1.316	0.262
rel	0	-	-	-
syd	-5.390	1.2697	18.022	0.000

Table 4 The result of Wald test (q = 0.6, α=0.05)

Variable	Coefficient	Standard Error (S.E.)	Wald	Prob
intercept	-1.352	4.9052	0.076	0.785
pos	57.319	11.7496	23.798	0.000
neg	-40.801	19.5625	4.350	0.047
x1	-40.107	14.3860	7.772	0.010
x2	1.026	0.3484	8.680	0.007
x3	-101.188	23.2071	19.011	0.000
x4	15.033	20.4543	0.540	0.469
x5	70.630	25.1919	7.861	0.009
x6	29.294	3.2642	80.534	0.000
s1	10.122	1.4271	50.310	0.000
s2	14.067	6.5063	4.675	0.040
s3	3.595	6.2236	0.334	0.568
s4	36.912	6.1706	35.783	0.000
s5	0	-	-	-
f1	-9.095	5.4020	2.835	0.104
f2	10.850	3.0301	12.821	0.001
pro	5.752	2.3674	5.904	0.022
ava	-3.267	4.2303	0.597	0.447
rel	0	-	-	-
syd	-8.178	1.1812	47.939	0.000

Supplementary Tables 2-4. The results of the quasi-likelihood ratio test, the goodness of fit test, Wald test are shown in Tables 2-4. The value of goodness of fit ranges from 0 to 1. The closer the goodness of fit is to 1, the better the model fits. The closer the goodness of fit is to 0, the worse the model fits. It can be found that the goodness of fit is less than 0.5 at 0.4, 0.15, 0.8 quantiles, and the model fits poorly. The goodness of fit ranges between 0.5 and 0.6 when the quantiles are 0.1, 0.85, 0.95, and the fitting effect of the model is general. The goodness of fit at other quantiles is above 0.6 and the fitting effect of model is good. For the quasi-likelihood ratio test, we observe that P-values are greater than 0.05 when quantiles are 0.1, 0.15, 0.4, 0.45, 0.85, 0.9, 0.95 at the significance level of 0.05 (α= 0.05), which means they fail the quasi-likelihood ratio test. While P-values are less than 0.05 at other quantiles, indicating they pass the quasi-likelihood ratio test. Wald test is often used to test the validity of explanatory variables. Except for the poor performances of Wald test at 0.1, 0.15, 0.85, 0.9, 0.95 quantiles with more than half of the explanatory variables failed the Wald test, the rest of the variables at other quantiles passed the Wald test. The results of Wald test when quantiles are 0.2, 0.6 are shown here.

Table 5 User requirements are embodied

User needs	Major performance	Keyword

Performance	Memory optimization；	Memory; Installation package
reliability	Subjective experience better；	Pay; privacy; Account
availability	function stability;	Froze; Back; Flash; Black
differentiated	small difference.	Dark mode; android; IOS;
functional deficiency	functional completeness.	Beautify; Dark mode; Group;
functional insufficiency	Functional friendliness and	QQ; Video time; document

Supplementary Table 5. User needs based on online comment mining include performance, reliability, availability, differentiated requirements of different systems, functional deficiency, and functional insufficiency. The main requirements of users in each part are shown in Table 5.
